# ﻿Morphological and molecular analyses reveal two new species of *Gibellula* (Cordycipitaceae, Hypocreales) from China

**DOI:** 10.3897/mycokeys.90.83801

**Published:** 2022-06-02

**Authors:** MingJun Chen, Ting Wang, Yan Lin, Bo Huang

**Affiliations:** 1 Anhui Provincial Key Laboratory for Microbial Pest Control, Anhui Agricultural University, Hefei 230036, China Anhui Agricultural University Hefei China

**Keywords:** Araneogenous fungi, Cordycipitaceae, spider, Taxonomy

## Abstract

*Gibellulapenicillioides***sp. nov.** and *G.longispora***sp. nov.**, two new species parasitising spiders collected in China, are illustrated and described, based on morphological features and multiloci phylogenetic analysis. The *G.penicillioides***sp. nov.** group is sister to the *G.scorpioides* group, but form long penicilloid conidiophore producing enlarged fusiform conidia ((7–) 7.5–9 (–10) × 2.5–3.5 μm). *G.longispora***sp. nov.** is sister to *G.pigmentosinum*, but has slender long conidia (5–7 × 1–2 μm); teleomorph and Granulomanus-synanamorphic conidiogenous cells are absent in these two species. Type specimens of *G.penicillioides***sp. nov.** and *G.longispora***sp. nov.** were deposited in the Anhui Agricultural University (RCEF). In addition, a key to all known species of Gibellula is illustrated.

## ﻿Introduction

Spider–pathogenic fungi, also called araneogenous or araneopathogenic fungi, are the group that infect spiders (phylum Arthropoda, class Arachnida, order Araneae) and belong to the Hypocreales (Evans and Samson 1987). About 91 Hypocrealean spider- and harvestman-pathogenic fungi were recognised to accommodate the genera *Akanthomyces* Lebert, *Beauveria* Vuill., *Clonostachys* Corda, *Cordyceps* Fr., *Engyodontium* de Hoog, *Gibellula* Cavara, *Hevansia* Luangsa-ard, Hywel-Jones & Spatafora, *Hirsutella* Pat., *Hymenostilbe* Petch, *Lecanicillium* W. Gams & Zare, *Ophiocordyceps* Petch, *Purpureocillium* Luangsa-ard, Hywel-Jones, Houbraken & Samson and *Torrubiella* Boud. (Shrestha et al. 2019). Of the above genera, only *Gibellula* and *Hevansia* are exclusively spider–pathogenic and present host specificity (Shrestha et al. 2019; Kuephadungphan et al. 2020). *Gibellula* species are amongst the most common spider pathogens in the world and are distributed from temperate to subtropical and tropical regions. Morphologically, the group can produce cylindrical synnemata from the outer loose hyphae covering spider cadavers with conidiophores abruptly narrowing to a short distinct neck and forming a subsphaeroidal vesical (Mains 1950; Samson and Evans 1992; Kuephadungphan et al. 2019).

In 1894, the genus *Gibellula* was proposed by Cavara (1894), based on *Gibellulapulchra* (Sacc.) Cavara (*Corethropsispulchra* Sacc.). Since then, many new taxa of parasitic *Gibellula* (mostly on spiders) have been described. Petch (1932) and Mains (1949, 1950) treated a number of *Gibellula* species as synonyms of *G.pulchra* and recognised only four species in the genus *Gibellula*. Kobayasi and Shimizu (1976, 1982) revised some of the existing species of *Gibellula* and described two new taxa. In a phylogenetically-based nomenclature for Cordycipitaceae (Hypocreales), all *Gibellula* samples fell into a single clade in the Cordycipitaceae; therefore, the genus *Gibellula* was revised and recognised as spider pathogens that produce synnemata with swollen conidiophores reminiscent of *Aspergillus* (Kepler et al. 2017). Recently, current nomenclature, diversity and distributions of *Gibellula* were reviewed and seventeen *Gibellula* species were recognised (Shrestha et al. 2019). Since then, five new species were described (Kuephadungphan et al. 2020; Chen et al. 2021): *G.cebrennini* Tasan., Kuephadungphan & Luangsa-ard, *G.fusiformispora* Tasan., Kuephadungphan & Luangsa-ard, *G.pigmentosinum* Tasan., Kuephadungphan & Luangsa-ard, *G.scorpioides* Tasan., Khons., Kuephadungphan & Luangsa-ard and *G.flava* Ming J. Chen & B. Huang. In all, we consider the genus *Gibellula* to include 22 species.

We carried out a series of collection trips for insect and spider pathogenic fungi in the Guniujiang National Forest Park in Anhui Province, China beginning in 2020. A total of seven spider cadavers infected by *Gibellula* were collected. One was identified as *G.flava* and four were similar to *G.scorpioides* in having solitary whip-like synnemata arising from host abdomens and penicillately-arranged conidiogenous cells. However, the four differed from *G.scorpioides* in having much longer synnemata and conidiophores and, thus, are here described as a new species, *G.penicillioides*. Three specimens from Nanling Nature Reserve, Guangdong Province were also identified as this new species through combined morphological and sequence data. We also found two collections similar to *G.pigmentosinum*, but with long and thin fusiform conidia. Due to these differences, we also describe them as a new species, *G.longispora*. Two additional specimens from Shenzheng, Guangdong Province were recognised as *G.longispora*. Multi-gene phylogenetic trees from these sampled fungi confirm their taxonomic placements. Here, we describe these two new species, distinguish them morphologically and phylogenetically and compare them with closely-related species.

## ﻿Materials and methods

### ﻿Sample collection and morphology

We collected five *Gibellula* samples from Guniujiang National Forest Park, Anhui Province, two samples from Shenzhen City, Guangdong Province and three samples from Nanling National Nature Reserve, Guangdong Province. The collections were carefully deposited in plastic boxes and returned to the laboratory. Microscopic observations were made from squash mounts and sections made from fresh material. The fresh structures were mounted in water for measurements and lactophenol cotton blue solution for microphotography, following Kuephadungphan et al. (2020). We observed microscopic characteristics, such as size and shape of conidia, phialide, vesicles, metulae and conidiophores using a ZEISS Axiolab 5 microscope. All samples studied here were deposited in the Research Center for Enotomogenous Fungi of Anhui Agricultural University (**RCEF**).

### ﻿DNA extraction, PCR amplification and sequencing

Total genomic DNA was extracted from fresh synnema with a modified CTAB method (Spatafora et al. 1998). Two gene portions from cell nuclei and three protein coding genes were used in this study: small subunit ribosomal RNA (SSU), large subunit ribosomal RNA (LSU), elongation factor-1a (TEF) and the largest and second largest subunits of RNA polymerase II (RPB1 and RPB2). SSU with NS1 and NS2 (White et al. 1990), LSU was amplified with primers LR0R and LR5 (Rehner and Samuels 1994), TEF-1 with TEF1–983F and TEF1–2218R (Rehner and Buckley 2005), RPB1 with CRPB1and RPB1–Cr (Castlebury et al. 2004) and RPB2 with fRPB2–7CR and fRPB2–5 (Liu et al. 1999). PCR amplification of the five nuclear loci was performed according to Kuephadungphan et al. (2019). PCR products were purified and sequenced by Sangon Company (Shanghai, China). The resulting sequences were checked manually before submission to GenBank.

### ﻿Sequence alignment and phylogenetic analysis

We constructed a phylogenetic tree using the five loci (SSU, LSU, TEF, RPB1 and RPB2) from 50 taxa (Table 1) within the Cordycipitaceae (Hypocreales). Multiple sequence alignment was performed with Clustal X (version 2.0) (Larkin et al. 2007) and manual adjustments of sequences were done using BioEdit, adjusted to maximise homology. All loci were subsequently concatenated using PhyloSuite v1.2.1 (https://github.com/dongzhang0725/PhyloSuite). The alignment was deposited at TreeBase (No. S29496).

**Table 1.** Accession numbers, strain numbers, and origins of *Gibellula* and related taxa used in this study, new sequences were shown in bold.

Taxon	Specimen vouchera	GenBank accession nos				
		SSU	LSU	*TEF*	RPB1	RPB2
*Akanthomycesaculeatus*	TS772	EU369110	KC519370	–	–	–
*A.aculeatus*	HUA 186145T	MF416572	MF416520	MF416465	–	–
*Beauveriabassiana*	ARSEF 7518	–	–	HQ880975	HQ880834	HQ880906
*B.bassiana*	ARSEF 1564T	–	–	HQ880974	HQ880833	HQ880905
*Cordycepsmilitaris*	OSC 93623	AY184977	AY184966	DQ522332	DQ522377	AY545732
*C.nidus*	TS903C	KY360300	KY360293	–	KY360296	–
*C.caloceroides*	MCA 2249	MF416578	MF416578	MF416525	MF416470	MF416632
*Blackwellomycescardinalis*	OSC 93609T	AY184973	AY184962	DQ522325	DQ522370	DQ522422
*B.cardinalis*	OSC 93610	AY184974	AY184963	EF469059	EF469088	EF469106
*Engyodontiumaranearum*	CBS 309.85	AF339576	AF339526	DQ522341	DQ522387	DQ522439
*E.aranearum*	CBS 658.80	–	LC092916	–	–	–
*Gibellulacebrennini*	BCC 39705	–	MH394673	MH521895	MH521822	MH521859
*G.cebrennini*	BCC 53605T	–	MT477062	MT503328	MT503321	MT503336
*G.clavulifera var. alba*	ARSEF 1915T	DQ522562	DQ518777	DQ522360	DQ522408	DQ522467
*G.flava*	WFS09061701	–	GU827389	–	–	–
*G.flava*	WFS20190625-25	MW036749	MW084343	MW091325	MW384883	–
*G.fusiformispora*	BCC 56802T	–	MT477063	MT503329	MT503322	MT503337
*G.fusiformispora*	BCC 45076	–	–	–	MH521823	MH521860
*G.gamsii*	BCC 27968T	–	MH152539	MH152560	MH152547	–
*G.gamsii*	BCC 28797	–	MH152541	MH152562	MH152549	MH152557
*G.leiopus*	BCC 16025	MF416602	MF416548	MF416492	MF416649	–
***G.longispora***	NHJ 12014	EU369098	–	EU369017	EU369055	EU369075
***G.longispora***	**GNJ20200813–16**	–	–	**MW961414**	**MW980145**	–
***G.longispora***	**GNJ20210710-02**	**OL854201**	**OL854212**	**OL981628**	–	**OL981635**
***G.longispora***	**SZ20210904-02**	–	–	**OL981630**	–	–
***G.longispora***	**SZ20210915-01**	–	–	**OL981631**	–	–
*G.pigmentosinum*	NHJ 11679	–	–	EU369016	EU369054	–
*G.pulchra*	GNHJ 10808	EU369099	EU369035	EU369018	EU369056	EU369076
*G.pigmentosinum*	BCC 41203T	–	–	MT503330	MT503323	–
*G.pigmentosinum*	BCC 39707	–	MH394674	MH521894	MH521801	MH521856
*G.scorpioides*	BCC 47976T	–	MT477066	MT503335	MT503325	MT503339
*G.scorpioides*	BCC 47530	–	MT477065	MT503334	–	MT503338
*G.scorpioides*	BCC 47514	–	–	MT503333	–	–
*G.scorpioides*	BCC 43298	–	MH394677	MH521900	MH521816	MH521858
*G.scorpioides*	BCC 13020	–	MH394686	MH521901	MH521814	–
*Gibellula sp.*	NHJ 7859	EU369107	–	–	EU369064	EU369085
*Gibellula sp.*	NHJ 10788	EU369101	EU369036	EU369019	EU369058	EU369078
*Gibellula sp.*	NHJ 5401	EU369102	–	–	EU369059	EU369079
***G.penicillioides***	**GNJ20200814–11**	**MW969669**	**MW969661**	**MW961415**	**MZ215998**	–
***G.penicillioides***	**GNJ20200814–14**	**MW969670**	**MW969662**	**MW961416**	**MZ215999**	–
***G.penicillioides***	**GNJ20200814–17**	**MW969671**	**MW969663**	**MW961417**	–	–
***G.penicillioides***	**GNJ20200812–05**	**MW969672**	**MW969664**	**MW961418**	–	–
***G.penicillioides***	**NL20210822-01**	–	–	**OL981632**	–	–
***G.penicillioides***	**NL20210822-09**	–	–	**OL981633**	–	–
***G.penicillioides***	**NL20210822-20**	–	–	**OL981634**	–	–
*Hevansiacinerea*	NHJ 3510	EU369091	–	EU369009	EU369048	EU369070
*H.novoguineensis*	CBS 610.80T	–	MH394646	MH521885	–	MH521844
*H.novoguineensis*	NHJ 11923	EU369095	EU369032	EU369013	EU369052	EU369072
*H.novoguineensis*	BCC 47881	–	MH394650	MH521886	MH521807	MH521845

References: (Sanjuan et al. 2014; Kepler et al. 2017; Rehner et al. 2011; Spatafora et al. 2007; Luangsa-ard et al. 2005; Helaly et al. 2019; Sung et al. 2007; Sung et al. 2001; Johnson et al. 2009; Kuephadungphan et al. 2020; Chirivi-Salomon et al. 2015; Kepler et al. 2012; Sung and Spatafora 2004; Tsang et al. 2016; Kuephadungphan et al. 2019; Helaly et al. 2017)

Phylogenetic inference was done according to Maximum Likelihood (ML) using RAxML 7.2.8 (Stamatakis 2006) and Bayesian Inference (BI) using MrBayes 3.3.7 (Ronquist and Huelsenbeck 2003). For the ML analysis, we used the GTRCAT model for all partitions, in accordance with recommendations in the RAxML manual against the use of invariant sites and 1000 rapid bootstrap replicates. The GTR+I+G model was selected by MrModeltest 2.2 (Nylander 2004) as the best nucleotide substitution model for the Bayesian analysis. Four MCMC chains were executed simultaneously for 2000,000 generations, sampling every 100 generations. Finally, phylogenetic trees were visualised using the Interactive Tree of Life (iTOL) (https://itol.embl.de) online tool (Letunic and Bork 2016).

## ﻿Results

### ﻿Taxonomy

#### ﻿ Gibellula penicillioides

Taxon classificationFungiHypocrealesCordycipitaceae

Ming J. Chen & B. Huang, sp. nov.

32C40875-F1E0-59B8-91CC-48640A867AA0

843174

Fig. 1

##### Etymology.

Latin “*penicillioides*” referring to the fungus with penicillate conidiophores.

##### Type.

China. Anhui Province: Shitai County, Guniujiang National Nature Reserve, on a spider, on unidentified leaf, 1 August 2020, Mingjun Chen & Bo Huang, holotype GNJ20200814-14. GenBank sequence data for GNJ20200814-14: SSU = MW969670; LSU = MW96966; TEF = MW961416; RPB1 = MZ215999.

##### Description.

**Figure 1.**
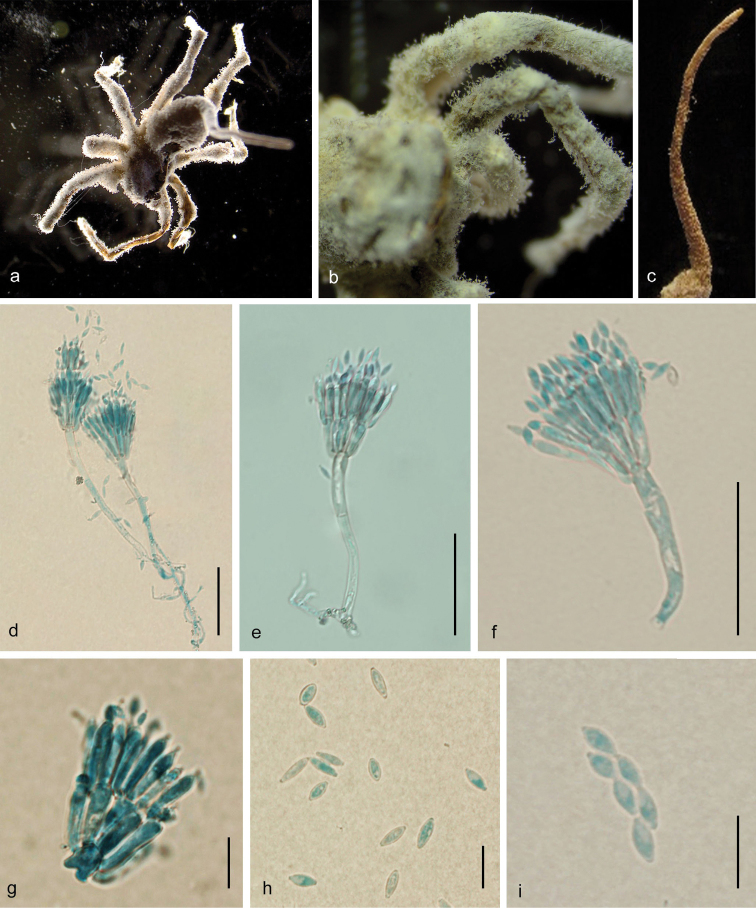
*Gibellulapenicillioides* sp. nov. **a–b** fungus on spider **c** synnema solitary **d–f** Penicillate conidiophores **g** conidiophore head bearing conidia **h** conidia **i** conidia in chains. Scale bars: 50 μm (**d, e, f**); 10 μm (**g, h, i**).

Mycelium covering the host, brownish–white cream–yellow to light–brown mycelial mat. Light greyish-brown to violaceous-brown when dried. Synnema solitary, white to yellowish, arising from the tip of the host’s abdomen, slender, cylindrical, 6.8 mm long, 0.6 mm wide at base and 0.1 mm at tip. Conidiophores rising from mycelial mat and synnema, smooth, septate, cylindrical, mostly biverticillate, (40–) 52.5–92 (115) × (4–) 4.5–6 μm (Fig. 1d, e), vesicles rarely developed. Several metulae are borne on the apex of conidiophore. Metulae clavate (slightly broadening towards the base) to cylindrical, (11–) 13–17.5 (21.5) × 3.5–5 (–5.5) μm, with a number of phialides in whorls. Phialides broadly cylindrical, with the apex tapering abruptly to a short neck (10–) 12.5–15.5 (–17) × (2.5–) 3–4 (–5) μm. Conidia fusiform, (7–) 7.5–9 (–10) × 2.5–3.5 μm, in chains, borne on each phialide (Figs 1i–j). Teleomorph and granulomanus synanamorphs not observed.

##### Habitat.

Occurring on spider attached to the underside of unidentified leaves nearby rivers.

##### Additional materials examined.

China. Anhui Province: Shitai County, Guniujiang National Nature Reserve, on a spider, 1 August 2020, Mingjun Chen & Ting Wang, GNJ20200814–11, GNJ20200814–17 and GNJ20200812–05. China. Guangdong Province: Nanling Nature Reserve, August 2021, on a spider, Qianle Lu, NL20210822-01, NL20210822-09, and NL20210822-20.

##### Notes.

In its morphological characters, *G.penicillioides* resembles *G.scorpioides*, *G.dabieshanensis* B. Huang, M.Z. Fan & Z.Z. Li, G. *clavulifera* var. clavulifera (Petch) Samson & H.C. Evans, G. *clavulifera* var. major Tzean, L.S. Hsieh, J.Y. Liou & W.J. Wu and G.clavuliferavar.alba Humber & Rombach by single synnema producing smooth penicillate conidiophores. Table 2 provides a comparative summary of the main characters of *G.penicillioides* and the other four species. Microscopically, *G.penicillioides* can be distinguished from *G.scorpioides*, *G.dabieshanensis* and G.clavuliferavar.clavulifera by having longer conidiophores and slightly larger conidia. Furthermore, *G.penicillioides* differs from G.clavuliferavar.alba by forming larger metulae, phialides and conidia, while G.clavuliferavar.major produces the largest conidia and the longest conidiophore.

**Table 2.** Comparison of *Gibellulaclavulifera*, *G.dabieshanensis*, *G.scorpioides and G.penicillioides* sp. nov. with penicillate conidiophores.

Species	Conidiophore(μm)	metulae (μm)	Phialide (μm)	Conidia (μm)
***Gibellulapenicillioides* sp. nov.^1^**	penicillate, smooth, mostly biverticillate or terverticillate, (40–) 52.5–92 (115) × (4–) 4.5–6	obovoid to cylindrical, (11–) 13–17.5 (21.5) × 3.5–5 (–5.5)	broadly cylindrical, (10–) 12.5–15.5 (–17) × (2.5–) 3–4 (–5)	(7–) 7.5–9 (–10) × 2.5–3.5
***Gibellulaclavulifera* var. major^2^**	penicillate, Smooth‐walled, mostly bi- or terverticillate, occasionally monoverticillate 140 × 4.8-7.1	clavate to cylindrical, 12.7-19.8 × 4.0-5.6	ampulliform to cylindrical, 12.7–19.8 × 3.6–4.8 (-5.3)	7.1–12.0 (–13.9) × 2.4–4.0 (–5.6)
**G*ibellula scorpioides*^3^**	penicillate, smooth, mostly biverticillate, 20–29 (–30) × 4	obovoid, slightly broadening toward the base, (7–) 9.5–12.5 (–15) × (2–) 3–5 (–7)	broadly cylindrical, (9–) 10–12.5 (–14) × (2–) 2.5–3.5 (–4)	5–7 (–9) × (1.5–) 2–3
**Gibellulaclavuliferavar.clavulifera ^4^**	penicillate, Smooth-walled, 45–50	clavate	cylindrical, with short neck 15–17.3 × 3.2–4.3	5.4–7.6 × 2.1–3.2
**Gibellulaclavuliferavar.alba ^5^**	penicillate, smooth, mono-or biverticillate, up to 100	cylindricrical, 9–15 × 3–4	cylindrical or slightly swollen near the middle 10–12.4 × 1.5–2.5	5–7.5 × 1.5–2
***Gibelluladabieshanensis***	penicillate with swollen vesicle, smooth 27–44	Obovoid to cylindricrical 8.6–11.5 × 5–6	cylindrical, 7.9-10.8 × 1.8-2.9	3.2-4.0 × 1.1-1.8

Note: ^1^Current study, ^2^Tzean et al. 1997, ^3^Kuephadungphan et al. 2020, ^4^Chen et al. 2014, ^5^Humber and Rombach 1987, ^6^Huang et al. 1998.

#### ﻿ Gibellula longispora

Taxon classificationFungiHypocrealesCordycipitaceae

Ming J. Chen & B. Huang, sp. nov.

461F4BC3-E70E-56CB-AF37-A87EAC7CF386

843175

Fig. 2

##### Etymology.

Latin “*longispora*” referring to the fungus with slender long conidia.

##### Type.

China. Anhui Province: Shitai County, Guniujiang National Nature Reserve, on a spider, on unidentified leaf, 1 August 2020, Mingjun Chen & Bo Huang, holotype GNJ20200813–16. GenBank sequence data for GNJ20200813–16: TEF = MW961414; RPB1 = MW980145.

##### Description.

**Figure 2.**
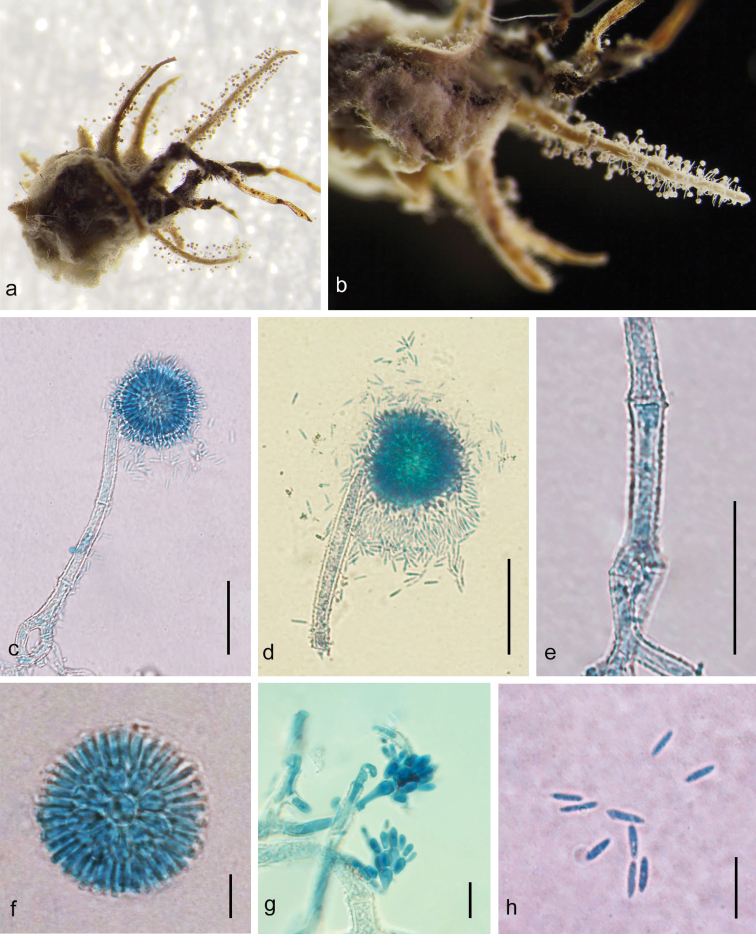
*Gibellulalongispora* sp. nov. **a, b** fungus on a spider **c, d** conidiophores showing conidial head **e** part of conidiophore showing rough walls **f, g** conidial head **h** conidia. Scale bars: 50 μm (**c, d**); 20 μm (**e**), 10 μm (**f, g, h**).

Mycelium covering the host, white to cream fluffy, light greyish-brown to violaceous-brown when dried. Synnema multiple, cylindrical, growing from abdomen of host spider, cream to yellowish–white. Conidiophores, (19-) 60-153.5 (-170) × 8–10 μm (Fig. 2d), crowded, lately arising from hyphae loosely attached to the surface of the synnema, verrucose, multiseptate, suddenly narrowing to a tip, then forming a globose vesicle, (5.5–) 6–8.5 (–9.5) × (5–) 5.5–8μm (Fig. 2c, f). Spherical conidial heads consisting of vesicle, metulae and phialide, (25.5–) 38.5–49 (–50) × (24.5) 36–46.5 (–49) μm. A number of broadly obovate to oval metulae, 6.5–9.5 × (4.5–)5–7 μm (Fig. 2c), borne on vesicle, each metulae bearing several clavate phialides, (6.5–) 7–9.5 (–11) × (1.5–) 2–3 μm (Fig. 2c, f). Conidia, 5–7 × 1–2 μm (Fig. 2g), narrowly fusiform. Teleomorph and granulomanus synanamorphs not observed. (Fig. 2f).

##### Habitat.

Occurring on spider attached to the underside of leaf nearby the river.

##### Additional materials examined.

China. Anhui Province: Shitai County, Guniujiang National Nature Reserve, on a spider, 10 July 2020, Mingjun Chen & Ting Wang, GNJ20210710-02. China. Guangdong Province: Shenzhen, 10 October 2021, on spiders, Qianle Lu, SZ20210904-02, and SZ20210915-01.

##### Note.

The new species *G.longispora* is similar to five *Gibellula* species in having multi-synnemum and aspergillate, distinctly roughened conidiophores (Table 3), namely *G.pigmentosinum*, *G.flava*, *G.pulchra*, *G.clavispora* Z.Q. Liang, Wan H. Chen & Y.F. Han and *G.shennongjiaensis* X. Zou, Wan H. Chen, Y.F. Han & Z.Q. Liang. However, *G.longispora* differs from *G.pigmentosinum*, *G.flava* and *G.pulchra* by its longer, slender conidia. Furthermore, compared to *G.longispora*, the species *G.shennongjiaensis* has shorter conidiophores with smaller phialide and metulae and slightly smaller conidia, while *G.clavispora* bears clavate conidia.

**Table 3.** Comparison of the morphological characters of *Gibellulalongispora* sp. nov. and related species.

Species	Conidiophore (μm)	Metulae (μm)	Phialide (μm)	Conidia (μm)
***Gibellulalongispora* sp. nov.*^1^***	verrucose, (19–) 60–153.5 (–170) × 8–10	obovoid to cylindrical, 6.5–9.5 × (4.5–) 5–7	clavate to broadly cylindrical, (6.5–) 7–9.5 (–11) × (1.5–) 2–3	fusiform, 5–7 × 1–2
***Gibellula pigmentosinum ^2^***	smooth to verrucose, (55–) 97.5–170 (–226) × (5–) 7–10 (–12.5)	broadly obovoid, (5.5–) 6–8 (–10) × (3–) 4–6 (–7.5)	obovoid to clavate, (5-) 5.5-8 (-9) × 2-3 (-4.5)	obovoid with an acute apex (2.5-) 3.5-5 (-5.5) × 1-2 (-3)
***Gibellulaflava ^3^***	verrucose, 33.5–123.5(–182.5) × (3–) 4–9.5 (–11.5)	obovoid to broadly obovoid, (4.5–) 5.5–7 × 3.5–5.5	narrowly obovate to clavate, 5.5–7 × 1.5–2.5	fusiform, (2.5–) 3–4 (–5.5) × 1–2(–3)
***Gibellulapulchra ^4^***	verrucose, 155–170 × (6–) 7.5–10	cylindrical, 6.2–7.5 × 5	clavate, 7.5–8 × 1.5–2.5	fusiform to fusiform-ellipsoid, 3–5 × 1.5–2.5
***Gibellulaclavispora ^5^***	smooth or occasionally roughened 96–113 long	obovoid, 8.6–10.8 × 2.2	clavate 5.4–6.5 × 1.1–2.2	clavate, single, 5.4–6.5 × 1.1–2.2
***Gibellulashennongjiaensis ^6^***	verrucose, 77–107 long	elliptical, 5.4–7.6 × 2.1–4.3	clavate,5.4–10.8 × 1.1–2.2	cylindrical or fusiform, 3.2–6.5 × 1.1–1.6

Note: ^1^Current study, ^2^Kuephadungphan et al. 2020, ^3^Chen et al. 2021, ^4^Chen et al. 2016, ^5^Faruk et al. 2004, ^6^Zou et al. 2016.

### ﻿Phylogenetic analysis

**Figure 3.**
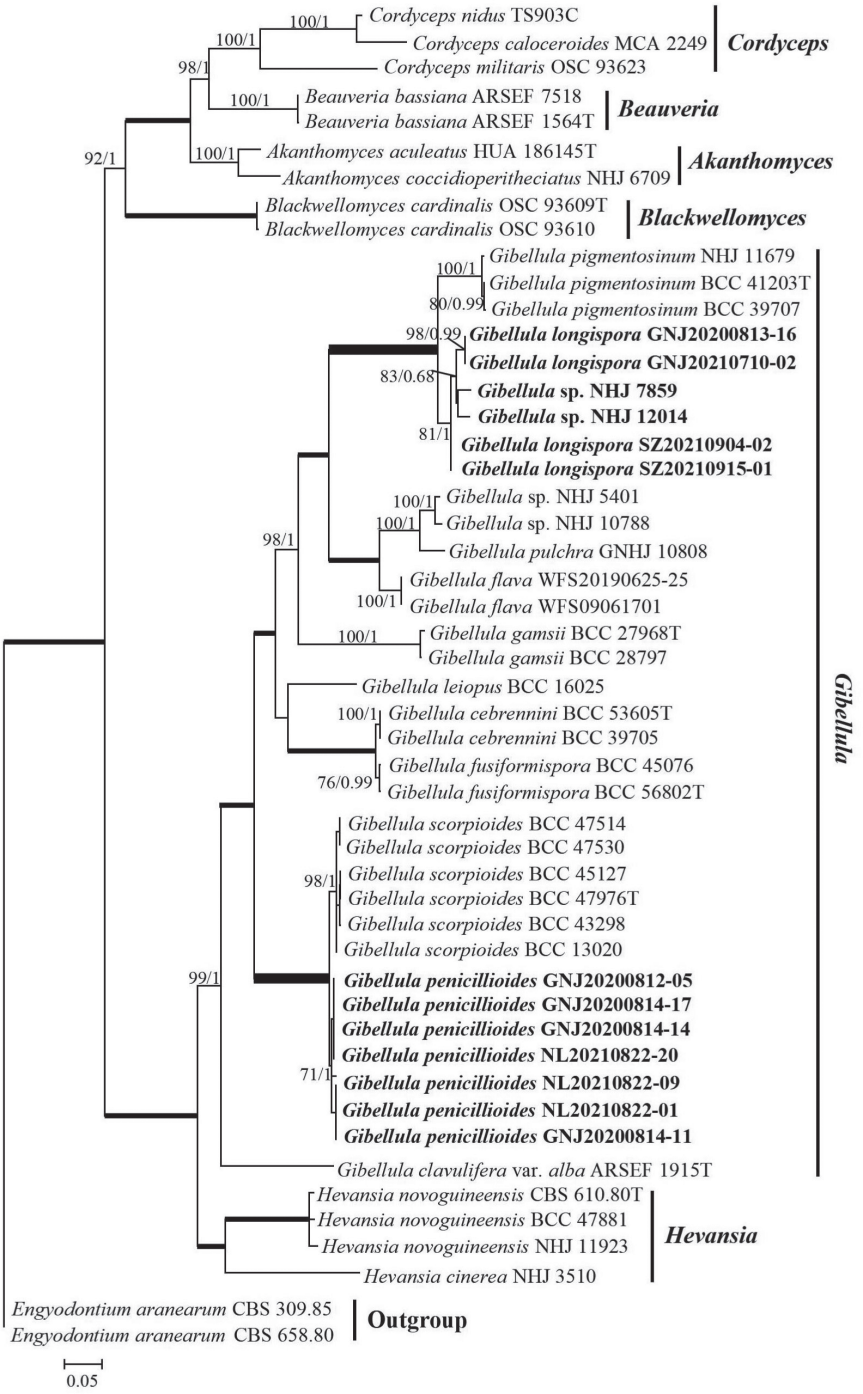
Phylogenetic relationships amongst *Gibellula* and related genera in Cordycipitaceae obtained from analyses of Maximum Likelihood (ML) analysis of five loci (SSU, LSU, TEF, RPB1 and RPB2). ML and BI topologies were generally congruent; therefore, we show only the ML analysis for brevity. At each node with support < 100%, we show ML bootstrap support / BI posterior probabilities; thick branches indicate 100% ML and BI support. The newly-proposed stains are highlighted in bold.

We constructed phylogenetic trees of the five concatenated loci from 11 newly-collected samples and 39 closely-related taxa from GenBank (Table 1). Our sampling included seven genera belonging to Cordycipitaceae, including *Akanthomyces*, *Beauveria*, *Blackwellomyces*, *Cordyceps*, *Engyodontium*, *Gibellula* and *Hevansia*, with *Engyodontiumaranearum* being used as the outgroup. The concatenated alignment was 4581 bases long, with 525 bases from SSU, 838 bases from LSU, 924 bases from TEF, 720 bases from RPB1 and 1056 bases from RPB2. The ML and BI phylogenic topologies were generally congruent (Fig. 3).

All *Gibellula* species, including the 11 new specimens, formed a monophyletic group with high support that was sister to *Hevansia*. Moreover, the seven samples (GNJ20200814–11, 20200814-14, 20200814–17, 20200812–05; NL20210822-01, 20210822-09, 20210822-20), newly described as *G.penicillioides*, formed a clade sister to *G.scorpioiodes*. The four *Gibellula* specimens, newly described as *G.longispora* (GNJ20200813–16, 20210710-02; SZ20210904-02, 20210915-01), formed a clade with two previous *Gibellula* collections (NHJ 12014, 7859) with posterior probability of 1% and 71% bootstrap support, respectively; this lineage was sister to *G.pigmentosinum*. Furthermore, a BLASTn search for homologues showed that the *Gibellula* GNJ20200813–16 TEF sequence had highest similarity to the corresponding sequence of *Gibellula* sp. (NHJ 12014) (99.33%), further supporting that all members of this lineage belong to *G.longispora*.

## ﻿Discussion

Our combined morphological and multilocus phylogenetic analyses distinguish *Gibellulapenicillioides* and *G.longispora* as new species, which we described and illustrated. We showed that *G.penicillioides* is sister to *G.scorpioides*, but forms long penicilloid conidiophores producing enlarged fusiform conidia ((7–) 7.5–9 (–10) × 2.5–3.5 μm) and that *G.longispora* is sister to *G.pigmentosinum*, but has slender long conidia (5–7 × 1–2 μm).

The fungal name *Gibellulalongispora* for isolate NHJ12014 was first proposed, based on phylogenetic analysis with SSU, TEF, RPB1 and RPB2 sequences, but without morphological description (Johnson et al. 2009). In GenBank, sequences of isolate NHJ12014 were recorded as an unidentified *Gibellula* isolate. Furthermore, the name *G.longispora* has not been recorded in the global fungal databases Index Fungorum (www.indexfungorum.org) or MycoBank (www.mycobank.org) (Kuephadungphan et al. 2020). Therefore, due to the lack of formal description of isolate NHJ12014, the species name *G.longispora* was an invalid publication in 2009. Our molecular phylogeny showed that the five specimens from China (GNJ20200813–16, GNJ20210710-02, NL20210822-20, SZ20210904-02 and SZ20210915-01) formed a clade with isolates NHJ12014 and NHJ 7859. The close phylogenetic relationship of these specimens suggests that they are conspecific despite the lack of morphological data for isolates NHJ12014 and NHJ 7859. Here, we described and illustrated the type specimen GNJ20200813–16 as a new species under the name *Gibellulalongispora*.

In China, spider-pathogenic fungi have been investigated for a long time, but until the 1980s, only one species (*G.pulchra*) was reported (Gao 1981). However, the first *Gibellula* species in China was misidentified and is actually *G.leiopus* (Vuill. ex Maubl.), mainly based on its very short conidiophore, which imparts a compact appearance. In the 1990s, three new *Gibellula* species and a new variety were described from Taiwan and Anhui Province. During the past decade, Zongqi Liang’s research group have carried out a comprehensive study of the taxonomy of *Gibellula* in China and proposed three new species and two Chinese new records. Recently, we also found and published a new *Gibellula* species with Torrubiella-like sexual morph. Overall, ten species or varieties have been reported in China (Kuephadungphan et al. 2020; Chen et al. 2021): *G.clavispora*, *G.clavulifera*, G.clavuliferavar.major, *G.curvispora* Y.F. Han, Wan H. Chen, X. Zou & Z.Q. Liang, *G.dabieshanensis*, *G.dimorpha* Tzean, L.S. Hsieh & W.J. Wu, *G.flava*, *G.leiopus*, *G.pulchra*, *G.shennongjiaensis* and *G.unica* L.S. Hsieh, Tzean & W.J. Wu. *G.pulchra* and *G.leiopus* are commonly distributed spider pathogenic fungi in southern China. The specimens used in this study were collected from Anhui and Guangdong Provinces, which suggests that the two new species may be widely distributed in southern China.

Kuephadungphan et al. (2020) indicated that host specificity can be used to assess the virulence and potential of biocontrol agents. Mycologists are increasingly interested in exploiting *Gibellula* fungi for bioactive compounds. For example, EPF083CE extracted from *G.pulchra* EPF083 was shown to be a new effective antimicrobial compound (Kuephadungphan et al. 2013). Pigmentosins A and B have been isolated from the spider–associated fungus *G.pigmentosinum* (Helaly et al. 2019) and two secondary metabolites, named gibellamines A and B, have been extracted from *G.gamsii* Kuephadungphan, Tasan. & Luangsa-ard (Kuephadungphan et al. 2019). Interestingly, pigmentosin B and gibellamines are specific to *G.pigmentosinum* and *G.gamsii*, respectively and these specialised compounds may be used as markers for the species’ chemical taxonomy (Kuephadungphan et al. 2020).

*Gibellula* is characterised by its specialised growth requirements; it is very hard to establish in culture (Samson and Evans 1973). Fortunately, the new taxon *G.penicillioides* was successfully isolated from conidia on the standard medium of potato dextrose agar (PDA), although the isolates grew slowly. In the future, we may be able to take advantage of *Gibellula* culture to explore more useful bio-active secondary metabolites or chemotaxonomic markers.

### ﻿Key to the species of *Gibellula*

**Table:** 

1	Conidiophores smooth-walled, mononematous or synnematous	**2**
–	Conidiophores typically rough-walled, mostly synnematous	**8**
2	Conidiophores strictly mononematous, with abruptly narrowing apex and vesicle	***G.mainsii***
–	Conidiophores mononematous or synnematous; typically penicillate	**3**
3	Conidiophores mononematous or synnematous, teleomorph absent or present	**4**
–	Conidiophores strictly mononematous, hyaline; teleomorph *Torrubiellaratticaudata*	**G.clavuliferavar.alba**
4	Conidiophores > 90 μm long; conidia large	**5**
–	Conidiophores < 50 μm long; conidia small	**6**
5	Granulomanus synanamorph present	**G.clavuliferavar.major**
–	Granulomanus synanamorph absent	***G.penicillioides***
6	Conidial heads purple, teleomorph absent	**G.clavuliferavar.clavulifera**
–	Conidial heads colourless, teleomorph present	**7**
7	Vesicle swollen; conidia 3.2–4.0 × 1.1–1.8 μm	***G.dabieshanensis***
–	Vesicles absent or hardly developed; conidia 5–7(–9) × (1.5–)2–3 μm	***G.scorpioides***
8	Synnemata single or double	**9**
–	Synnemata multiple	**16**
9	Synnemata terminating in a bulbous outgrowth from which a number of conidiophores and a typical wing-like structure arise	***G.alata***
–	Synnemata not terminating in a bulbous outgrowth with a wing-like structure, but cylindrical, clavate or bulb-shaped	**10**
10	Synnemata typically club-shaped or clavate with a cylindrical sterile apical projection	**11**
–	Synnemata cylindrical without a sterile apical projection	**13**
11	Synnemata typically club-shaped; conidiophores > 80 μm long	***G.mirabilis***
–	Synnemata clavate; conidiophores < 80 μm long	**12**
12	Granulomanus synanamorph present	***G.clavata***
–	Granulomanus synanamorph absent***G.gamsii***	
13	Granulomanus synanamorph present	**14**
–	Granulomanus synanamorph absent or occasionally present	**15**
14	Granulomanus synanamorph with well-differentiated conidiophore and polyblastic conidiogenous cells	***G.dimorpha***
–	Granulomanus synanamorph with polyblastic conidiogenous cells	***G.cebrennini***
15	Conidiophore 97–170 μm long; conidia obovoid with an acute apex	***G.pigmentosinum***
–	Conidiophore 31–53 μm long; conidia fusiform to broadly fusiform	***G.fusiformispora***
16	Synnemata with a stout yellowish-tan stipe, broadening into globose to pyriform fertile area and narrowed into a pale brown compact acuminate sterile tip	***G.brunnea***
–	Synnemata cylindrical	**17**
17	Granulomanus synanamorph present	**18**
–	Granulomanus synanamorph absent	**19**
18	Granulomanus synanamorph with well-differentiated conidiophore and polyblastic conidiogenous cells	***G.unica***
–	Granulomanus synanamorph with polyblastic conidiogenous cells in culture	***G.shennongjiaensis***
19	Conidia clavate or botuliform	**20**
–	Conidia fusiform	**21**
20	Conidia 4.7–11 μm long, botuliform; Phialide globose in base	***G.curvispora***
–	Conidia 3.2–6.5 μm long, clavate; Phialide clavate	***G.clavispora***
21	Conidia > 5 μm long	***G.longispora***
–	Conidia < 5 μm long	**22**
22	Conidiophores long, with radiate and often loose conidial heads	**23**
–	Conidiophores short, with compact conidial heads	***G.leiopus***
23	Conidiophores up to 600 μm; conidia 3–5 μm in size	***G.pulchra***
–	Conidiophores up to 120 μm; conidia 3–4 μm in size	***G.flava***

## Supplementary Material

XML Treatment for Gibellula penicillioides

XML Treatment for Gibellula longispora
